# Urethral stricture in a 46,XX male with congenital adrenal hyperplasia (CAH) due to 21-hydroxylase deficiency: A literature review and case report

**DOI:** 10.1016/j.eucr.2025.103182

**Published:** 2025-08-27

**Authors:** Laura Gallardo Zamora, Yesica Quiroz Madarriaga, Anna Bujons Tur, Antoni Sanchez i Puy, David Salinas Duffo, Juan Antonio Peña Gonzalez

**Affiliations:** a Witten/Herdecke University, Witten, Germany; b Department of Urology, Uros Associats, Barcelona, Spain; c Department of Pediatric Urology, Fundacion Puigvert, Barcelona, Spain

**Keywords:** 46, XX male, Urogenital sinus, Urethral stricture, Urethral surgery

## Abstract

Disorders of sex development in 46,XX male individuals are rare and present complex surgical challenges, particularly regarding urethral stricture following urethral surgery. We present a 46,XX individual raised male who underwent early urethral surgery and developed a urethral stricture in adulthood. Surgical repair was not feasible due to anatomy and comorbidities. A systematic review on the management of urethral strictures in this patient population was conducted. Only one case of successful buccal mucosa graft urethroplasty in a 46,XX male was found. This case underscores the scarcity of data and challenges in managing urethral strictures in virilized 46,XX individuals.

## Highlights

• Urethral strictures in virilized 46,XX males are rare and complex to manage.
• Only three studies reported relevant cases, with limited surgical outcome data.
• One documented buccal graft urethroplasty showed successful short-term results.
• Open or robotic reconstruction is preferred for complex or obliterative strictures.
• More case reports and long-term data are needed to guide treatment protocols.

## 1 Introduction

46,XX males with CAH due to 21-hydroxylase deficiency are a rare population of individuals with DSD. Although genetically female, these individuals are exposed to high levels of androgens during the pregnancy, leading to virilization of external genitalia. Very few undergo masculinizing surgery early in life, including urethral surgery and genitoplasty. One of the most common long-term complications of such procedures is urethral stricture formation.^1^

Urethral stricture disease in 46,XX males presents diagnostic and surgical challenges due to anatomical complexity and prior surgeries. While urethral strictures are well-described in the general population, there is limited data regarding their management in virilized 46,XX individuals.^2^

This report presents a clinical case of severe urethral stricture in a virilized 46,XX male with CAH and includes a systematic review of surgical approaches to urethral strictures in this population.

## 2 Materials and methods

**Fig. 1:**
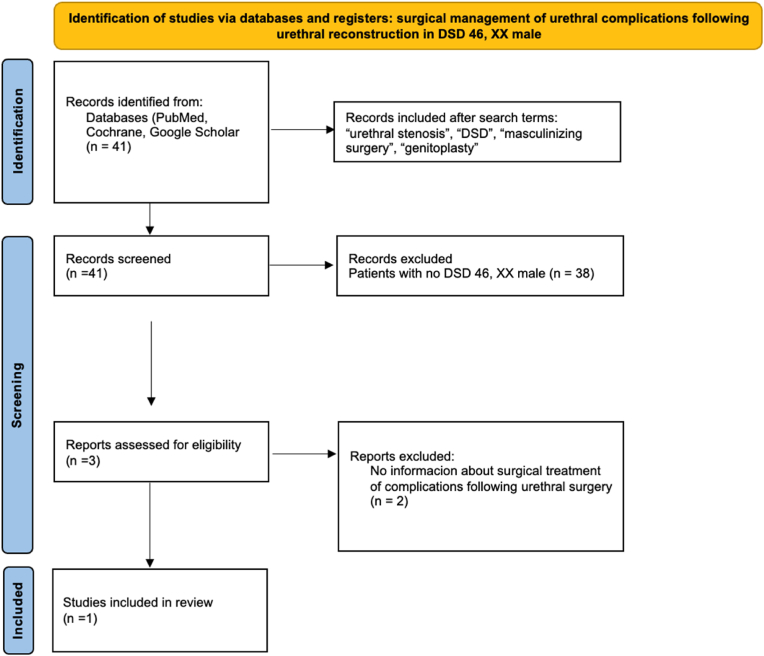
PRISMA flow diagram of surgical management of urethral complications following urethral reconstruction in DSD 46, XX male.

The PRISMA flow chart for systematic review is schematically reported in Fig. 1. Searches were conducted in PubMed, Cochrane Library, and Google Scholar using terms including “urethral stenosis,” “46,XX male,” “genitoplasty,” and “masculinizing surgery.” Only English-language studies from 1954 to September 2024 were included. Articles were screened manually, and reference lists were reviewed. Inclusion criteria required articles to report data on epidemiology, symptoms, diagnostic tools, surgical treatment, and follow-up of urethral strictures in virilized 46,XX patients. Duplicates and unrelated publications were excluded and data were extracted using a predefined form. The initial search identified 41 articles for urethral stenosis in DSD patients. After screening, only three studies^3^(2)^4^ were assessed for eligibility. Ultimately, two patients,^3^ including our own, had documented surgical treatment of urethral strictures in this particular population.

## 3 Results

In one case, a 26-year-old patient with 46,XX male syndrome presented with a perineal fistula. A ventral-onlay buccal mucosal graft urethroplasty was performed to address a long bulbar urethral stricture. This patient showed no recurrence during a one-year follow-up period, and sufficient data from retrograde urethrography and physical exams confirmed the success of the procedure.^3^

A larger study involving 25 patients (children, adolescents, and adults) who had undergone genitoplasty for DSD over a 14-year follow-up period identified common complications such as urinary fistulas and urethral strictures. However, there was no detailed data on the management or treatment of urethral strictures in these cases, indicating a lack of long-term treatment strategies for these complications.^2^

Finally, a report on surgical outcomes in children under two years old with moderate to severe genital atypia documented cases of meatal stenosis and urethral dehiscence. The follow-up period lasted one year, but again, there was no long-term data on stricture treatment or recurrence, emphasizing a consistent gap in the literature regarding the management of urethral strictures in this population.^4^

Therefore, among the articles assessed for eligibility, only one case report was identified that described a 46,XX male patient who underwent urethral surgery, with detailed documentation of the diagnosis, treatment, and one-year follow-up of complications following urethral surgery.^3^

## 4 Discussion

The reviewed cases emphasize the surgical complexity of managing urethral strictures in virilized 46,XX patients with a urogenital sinus. Previous surgeries, altered anatomy, and limited clinical guidelines contribute to these challenges. We present one clinical case to expand the literature on the management of urethral strictures in this patient population.

A patient born in 1944 presented with ambiguous genitalia and was assigned male at birth. At age four, he underwent urethal surgery due to hypospadias. At age six, bilateral testicular implants were placed following negative inguinal and abdominal exploration for testes. Testosterone was administered in adolescence to promote secondary male characteristics.

In 1982, karyotyping confirmed a 46,XX DSD with CAH due to 21-hydroxylase deficiency was diagnosed. Diagnostic laparoscopy and cystoscopy later confirmed bilateral tubal structures, a hypoplastic uterus, and a urogenital sinus.

In adulthood, the patient developed progressive voiding difficulties and recurrent febrile urinary tract infections, treated chronically with alpha-blockers. Eventually, he presented in septic shock due to acute urinary retention. Initial attempts at catheterization failed, likely due to a urethral stricture, following suprapubic catheter insertion.

**Fig. 2:**
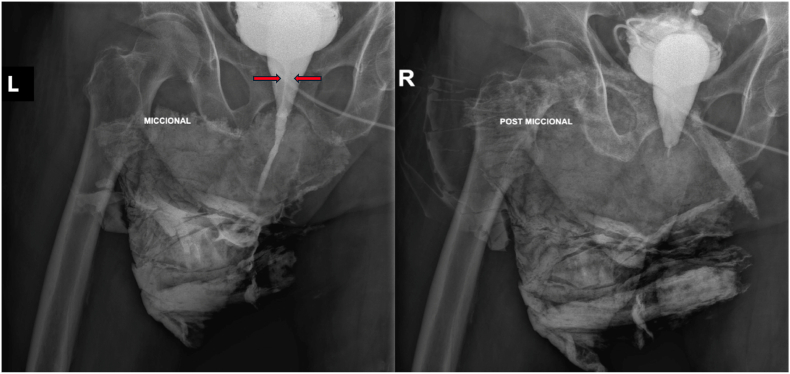
Voiding cystourethrography. *Left:* Voiding phase showing a urethral stricture (indicated between red arrows). *Right:* Post-void image.

**Fig. 3:**
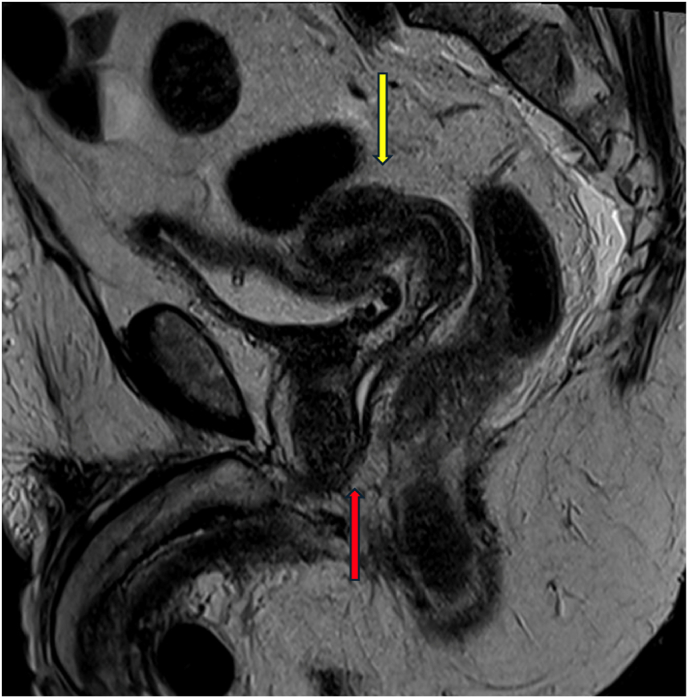
Pelvic magnetic resonance imaging showing uterus (yellow arrow) and urogenital sinus (red arrow).

**Fig. 4:**
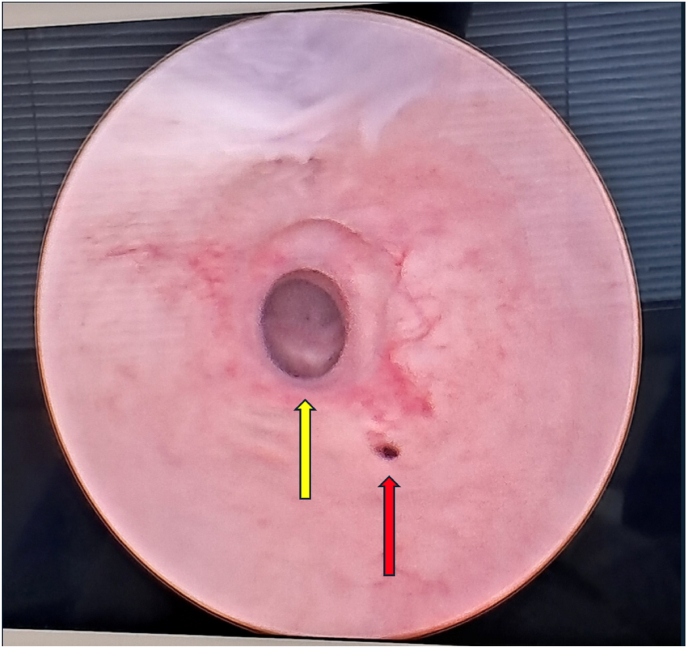
Endoscopic image of entrance to uterus (yellow arrow) and severe urethral stricture (red arrow).

As shown in Fig. 2, retrograde and antegrade cystography confirmed a long, severe posterior urethral stricture and the presence of a urogenital sinus. Fig. 3 further supports this finding with pelvic MRI imaging. Diagnostic cystoscopy was performed; however, it was not posible to insert a guide wire in the posterior urethra via either the antegrade or retrograde approach. Access to the urogenital sinus and a rudimentary uterus was achieved, as show in Fig. 4.

Given the patient's advanced age, anatomical complexity, and comorbidities, surgical options were limited. Long-term indwelling suprapubic catheterization was chosen for management.

Current options of treatment of posterior urethral strictures include endoluminal treatment for nonobliterative stenoses such as dilation, incision (cold/hot knife or Holmium laser), or resection. Success rates for first-time treatments vary from 50 % to 80 %.^5^ Injection of antifibrotic agents such as corticosteroids or mitomycin C may improve outcomes. However, recurrence remains common, and repeated interventions may be necessary. For obliterative strictures, open or robotic reconstruction is preferred due to higher failure and complication rates with blind endoscopic procedures.

Open surgical repair remains the gold standard for complex or recurrent posterior urethral strictures. Perineal, abdominal, or abdominoperineal approaches are chosen based on stricture location, length, and prior surgeries. Techniques include stepwise perineal excision and primary anastomosis, often requiring maneuvers like crural separation or pubectomy. Substitution urethroplasty with buccal mucosa grafts also offers good outcomes.^6^ Incontinence rates are higher after perineal versus retropubic approaches, and patients often require a second procedure to manage incontinence.^5^

Robotic-assisted surgery offers enhanced visualization and lower morbidity. Transabdominal or single-port transvesical techniques are emerging, with reported patency rates of 75–100 % and continence in 71–82 % of patients. Robotic approaches may reduce complications linked to open surgery, but expertise and careful patient selection are essential.^7^

In addition to the established surgical approaches, several less explored or palliative options have been reported. One such technique is transurethral incision with transverse mucosal realignment, which has demonstrated promising early results, including high patency rates and an absence of new-onset incontinence.^8^ However, further studies are needed to validate its long-term efficacy. Urethral stents, have shown short-term effectiveness but are now infrequently used due to complications like fibrosis and encrustation.^9^ Indwelling catheterization remains a palliative measure, generally reserved for patients with significant comorbidities or high operative risk. Although it offers immediate relief in cases of acute urinary retention, it is not a definitive treatment and is associated with risks such as recurrent infections and diminished quality of life.^10^

## 5 Conclusions

Urethral strictures in virilized 46,XX patients represent a rare but significant surgical challenge. Due to the limited data available in the literature, treatment decisions must be made on a case-by-case basis, considering the patient's anatomy, previous surgeries, and overall health. Through this systematic review and case report, we aim to contribute to the development of surgical protocols and improve the long-term management of urethral strictures in this unique population. The need for more comprehensive documentation and long-term follow-up in virilized 46,XX individuals is crucial to better understand and manage this condition.

## CRediT authorship contribution statement

**Laura Gallardo Zamora:** Conceptualization, Data curation, Formal analysis, Writing – original draft. **Yesica Quiroz Madarriaga:** Conceptualization, Methodology, Writing – review & editing. **Anna Bujons Tur:** Writing – review & editing. **Antoni Sanchez i Puy:** Writing – review & editing. **David Salinas Duffo:** Writing – review & editing. **Juan Antonio Peña Gonzalez:** Supervision, Writing – review & editing.

## References

[1] Gorduza D., Vidal I., Birraux J. (2010 Sep). The surgical challenges of disorders of sex development (DSD). Arch Esp Urol.

[2] Zhang H., Pan J., Ji H. (2013 Nov 25). Long-term evaluation of patients undergoing genitoplasty due to disorders of sex development: results from a 14-Year Follow-Up. Sci World J.

[3] Hosseini J., Zamani Hajiabadi A., Mirjalili A.M. (2023 Apr 10). Ventral-onlay buccal mucosal graft urethroplasty of a perineal fistula in a 26-Year-Old patient with 46 XX Male syndrome: a case report. Am J Mens Health.

[4] Bernabé K.J., Nokoff N.J., Galan D. (2018 Apr). Preliminary report: surgical outcomes following genitoplasty in children with moderate to severe genital atypia. J Pediatr Urol.

[5] Yu J., Ahmed M., Murray B., Ajay D. (2024 Sep). Posterior urethral stenosis: contemporary management options. UroPrecision.

[6] Bagga H.S., Angermeier K.W. (2015 Mar). The mechanism of continence after posterior urethroplasty. Arab J Urol.

[7] Unterberg S.H., Patel S.H., Fuller T.W., Buckley J.C. (2019 Mar). Robotic-assisted proximal perineal urethroplasty: improving visualization and ergonomics. Urology.

[8] Abramowitz D.J., Balzano F.L., Ruel N.H., Chan K.G., Warner J.N. (2021 Jun). Transurethral incision with transverse mucosal realignment for the management of bladder neck contracture and vesicourethral anastomotic stenosis. Urology.

[9] Cubuk A., Weinberger S., Moldovan E.D., Schaeff V., Neymeyer J. (2023 Sep). Use of the allium round posterior stent for the treatment of recurrent vesicourethral anastomosis stricture. Urology.

[10] Campos-Juanatey F., Osman N.I., Greenwell T. (2021 Aug 1). European association of urology guidelines on urethral stricture disease (part 2): diagnosis, perioperative management, and Follow-up in males. Eur Urol.

